# Comparative efficacy and safety of different treatment strategies for primary advanced ovarian cancer: a systematic review and network meta-analysis of randomized control trials

**DOI:** 10.3389/fonc.2026.1757826

**Published:** 2026-03-12

**Authors:** Yingfan Chen, Yuquan Yuan, Muheng Tao, Junlei Li, Xiaofan Zhu, Yiqing Xiong, Siqi Li, Chun Liu, Zheng Luo, Chengzhi Zhao, Cheng Chen

**Affiliations:** 1 Graduate School of Medicine, Chongqing Medical University, Chongqing, China; 2 Department of Obstetrics and Gynecology, Chongqing General Hospital, Chongqing University, Chongqing, China; 3 Department of Gynecologic Oncology, Chongqing Health Center for Women and Children, Chongqing, China; 4 Department of Obstetrics and Gynecology, Southwest Hospital, Army Medical University, Chongqing, China; 5 Peking University Chongqing Research Institute of Big Data, Chongqing, China

**Keywords:** adverse events, cytoreductive surgery, hyperthermicintraperitoneal chemotherapy, primary advanced ovarian cancer, survival outcomes

## Abstract

**Background:**

For patients with primary advanced ovarian cancer (OC), multiple treatment strategies based on cytoreductive surgery and adjuvant chemotherapy are available, but the optimal strategy remains undetermined. This network meta-analysis (NMA) aims to evaluate the efficacy and safety of primary debulking surgery (PDS), interval cytoreductive surgery (IDS) and IDS combined with hyperthermic intraperitoneal chemotherapy (HIPEC) in treating primary advanced OC.

**Methods:**

We conducted a comprehensive search of PubMed, EMBASE, Web of Science, and the Cochrane Library databases, covering publications up to June 23, 2025. The surface under the cumulative ranking curve (SUCRA) was used to assess the probability that each treatment strategy would be the better choice for each outcome.

**Results:**

Seven RCTs involving 2,058 patients were included in this NMA. Based on the SUCRA values, the IDS plus intraoperative HIPEC strategy achieved the highest ranking for overall survival (OS; SUCRA = 0.99) and disease-free survival (DFS; SUCRA = 0.99). Meanwhile, the addition of intraoperative HIPEC did not increase major postoperative complications (SUCRA = 0.68) or prolong the duration of hospital stay (SUCRA = 0.47). IDS could enhance the complete resection rate (SUCRA = 0.81) and reduce major postoperative complications (SUCRA = 0.75).

**Conclusions:**

Until new evidence becomes available, IDS plus intraoperative HIPEC are associated with better OS and DFS for patients with primary advanced OC. Besides, IDS had advantages in increasing the complete resection rate and reducing major postoperative complications.

## Introduction

Ovarian cancer (OC) is a common and fatal malignancy in women with high mortality (1). Due to the nonspecific symptoms in early stages and lack of specific biomarkers, over 70% of patients are diagnosed at advanced stages upon their first diagnosis (2). Over the past decades, although chemotherapy and targeted therapy are widely used, the prognosis of OC has remained unsatisfactory, with the five-year survival rate below 29% in advanced-stage patients (3).

Cytoreductive surgery (CRS) serves as the predominant clinical intervention for advanced OC (4). Primary debulking surgery (PDS) is the initial cytoreductive procedure performed prior to chemotherapy in patients diagnosed with OC, constituting a specific type of CRS. The latest National Comprehensive Cancer Network (NCCN) guideline recommends that patients with advanced OC who are deemed suitable for R0 resection based on preoperative imaging and can tolerate surgery should undergo PDS followed by platinum-based systemic adjuvant chemotherapy (5). Due to the widespread systemic metastases characterized by pelvic implantation dissemination in advanced OC, PDS may be intolerable or unable to achieve optimal tumor reduction. Research suggests that neoadjuvant chemotherapy (NACT) followed by interval cytoreductive surgery (IDS) and systemic adjuvant chemotherapy postoperatively is an alternative approach for patients with advanced OC after comprehensive assessment (6). Some evidence indicates that IDS can potentially improve the preoperative physical status of patients with advanced OC, reduce tumor size and extent, enhance the feasibility of complete resection, and thus elevate optimal cytoreduction rates (7, 8). Nevertheless, some studies argue that NACT appears not to provide additional survival benefit and may induce tumor fibrosis, increasing microscopic lesions that are not amenable to surgical resection, thus making complete cytoreduction more challenging (9, 10). Additionally, systemic chemotherapy drugs cannot sufficiently permeate peritoneal lesions due to the plasma-peritoneal barrier, resulting in limited effectiveness against microscopic residual tumors in the abdominal cavity. The use of NACT followed by IDS in advanced OC remains controversial.

With continuous exploration of OC treatments, CRS combined with hyperthermic intraperitoneal chemotherapy (HIPEC) has been proposed as a potential therapeutic strategy for improving clinical outcomes. On the one hand, intraperitoneal chemotherapy administration achieves higher drug concentrations in the peritoneum (11); on the other hand, during HIPEC therapy, heat exerts direct cytotoxic effects on tumor cells and enhances drug uptake through hyperthermia, thus strengthening treatment efficacy (12). Some studies have found that increasing intraoperative HIPEC can improve the survival condition of patients with advanced OC (13, 14). Emerging evidence proposes that combining HIPEC after CRS could improve outcomes by eliminating residual microscopic peritoneal disease (15, 16). PDS, IDS and IDS with intraoperative HIPEC exhibited distinct advantages and limitations for primary advanced OC. It is vital to investigate optimized and effective therapeutic methods to reduce complications and improve survival outcomes.

At present, systematic comparative studies evaluating efficacy and risks among different treatment strategies for primary advanced OC are limited and studies are mainly carried out through pairwise meta-analysis. Most of the meta-analysis include original studies that consist of retrospective or prospective research, and may introduce additional uncertainty (17, 18). In this study, we conducted a network meta-analysis (NMA) of randomized controlled trials (RCTs) to comprehensively evaluate the efficacy and safety of different treatment strategies for primary advanced OC. The goal was to provide more reliable evidence-based guidance for clinical practice.

## Methods

### Design and registration

This network meta-analysis reports the efficacy and safety of three different therapeutic strategies for primary advanced ovarian cancer based on the Preferred Reporting Items for Systematic Reviews and Meta-Analyses (PRISMA) guidelines (19). This study has been registered in the International Prospective Register of Systematic Reviews (PROSPERO CRD420251059337).

### Search strategy

The data used in this review came from the PubMed, Cochrane Library databases, EMBASE and Web of Science databases, based on the data collected on June 23, 2025. Medical Subject Headings (MeSH) and the free words of the keywords, including all known spellings of “Ovarian neoplasms”, “Hyperthermic intraperitoneal chemotherapy”, “Neoadjuvant chemotherapy” and “Cytoreduction Surgical Procedures” were searched from the above databases. In addition, the retrieved literature as well as reference lists of related meta-analyses and reviews were examined to maximize the comprehensiveness of the literature search.

### Inclusion and exclusion criteria

Studies were selected according to the following inclusion criteria: (1) Study design: only RCTs were included. (2) Population: women with newly diagnosed advanced ovarian cancer or tubal cancer (International Federation of Gynecology and Obstetrics (FIGO) stage III or IV). (3) Intervention: studies comparing at least two different kinds of treatment strategies, including PDS, IDS and IDS plus HIPEC. (4) Outcomes: studies reporting at least one outcome of interest mentioned below. (5) Language: studies that were published in English. Exclusion criteria were as follows: (1) Studies that were reviews, conference abstracts, guidelines, letters, editorials, case reports, etc. (2) Study population included recurrent OC. (3) Studies were not RCTs. (4) Studies with incomplete data or results reported with significant bias.

### Data extraction

Full-text review, quality assessment, and data extraction were independently conducted by two researchers (Y.F.C and Y.Q.Y). Any discrepancies were resolved by consulting a third reviewer (M.H.T) and conducting a thorough comparison of the data.

The extracted data mainly included the following: (1) Study information: first author, publication year, countries and study design. (2) Baseline characteristics: study population, age, FIGO stage, histological subtypes, NACT regimens and HIPEC regimens. (3) Outcome information: OS, DFS, rates of surgical cytoreduction achieving complete resection, operative time, duration of hospital stay and numbers of grade 3 and 4 adverse events occurring after surgery. When survival data could not be explicitly obtained, GetData Graph Digitizer v2.24 was used to extract data from Kaplan-Meier survival curves following the method of Tierney et al. The natural logarithm of HR (lnHR) and SE (standard error) were calculated for subsequent analysis (20).

### Quality assessment

Two review authors (Y.F.C and Y.Q.Y) independently assessed risk of bias in the included studies using Cochrane’s RoB 1 tool, with any differences of opinion resolved by discussion (21). Review Manager 5.3 was used to generate and display figures of the quality assessment results.

### Pairwise meta-analysis

Pairwise meta-analysis (PMA) involving at least two direct comparisons were conducted using R (version 4.3.1, RStudio, Boston, MA, USA). Dichotomous variables were measured using the odds ratio (OR) with a corresponding 95% CI, and continuous variables were analyzed using the mean difference (MD) with a corresponding 95% CI. Time-to-event outcomes (OS and DFS) were evaluated using hazard ratios (HRs) with 95% confidence intervals. We evaluated heterogeneity across studies using the Q test and I² statistic. If p ≤ 0.05 or I² ≥ 50%, significant heterogeneity was considered present and the random effects model was applied; otherwise, the fixed effects model was used. Forest plots were used to graphically present the combined effect estimates of the included studies. The Egger’s linear regression test was used to detect potential publication bias, with a p-value ≤ 0.05 indicating its presence.

### Methods of evidence synthesis in NMA

In the NMA, a Bayesian framework-based statistical model was built using JAGS version 4.3 with Markov Chain Monte Carlo (MCMC) methods in R (version 4.3.1, RStudio, Boston, MA, USA). The model fit between random effects and fixed effects models was compared using the Deviation Information Criterion (DIC), where a lower DIC indicates better model performance. In order to evaluate inconsistency, we plotted the posterior mean deviance contributions for each data point under both the consistency and inconsistency models (22). The surface under the cumulative rank curve (SUCRA) scores and mean ranks were used to determine the relative rankings of outcomes for the three advanced OC treatment strategies. The SUCRA values for each outcome under different treatment strategies were calculated. The SUCRA values closer to 1 indicate a higher probability that the treatment strategy is superior. NMA results were presented as median posterior mean differences (MD), odds ratios (OR), or hazard ratios (HR), along with their 95% credible intervals (CrIs). Data analysis was conducted using the “meta”, “gemtc” and “BUGSnet” packages in R statistical software (version 4.3.1).

## Results

### Study selection, sensitivity analysis, inconsistency and publication bias

**Figure 1:**
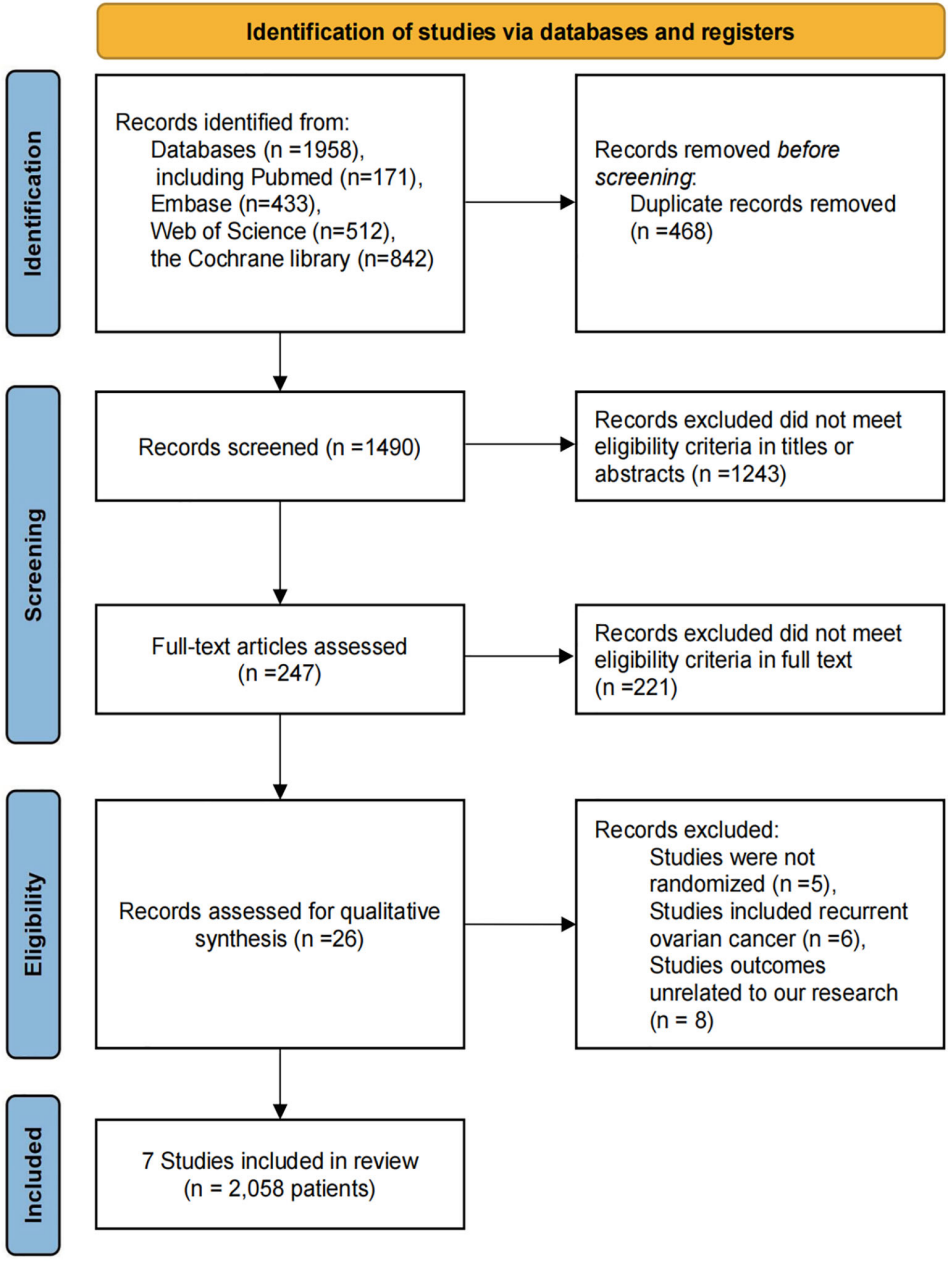
**A** PRISMA flowchart of searching and selecting eligible studies included in the network meta-analysis.

Using the predefined search strategy, 1,958 potentially relevant articles were identified across the databases. After excluding duplicate studies and full-text review, seven high-quality RCTs involving 2,058 patients were included in this study (15, 23–28). A PRISMA flowchart provides a visual representation of the selection and exclusion process in detail (**Figure 1**). Detailed information on RCT quality assessment is provided in **Supplementary Figure 1**.

The network relationships among treatment strategies for each outcome are shown in **Supplementary Figure 2**. The fit of random-effects and fixed-effects models for the NMA is presented in leverage plots (**Supplementary Figure 3**). No significant inconsistencies were observed across the outcomes of different treatment strategies in the NMA (**Supplementary Figure 4**). Publication bias in the included studies was assessed using calibration funnel plots and the Egger’s test. No significant evidence of publication bias was found, as depicted in **Supplementary Figure 5**.

### Study characteristics

This review incorporated seven RCTs conducted between September 2010 and September 2023, comprising six multicenter trials and one single-center trial. Overall, 2,058 patients with primary advanced OC were enrolled, with mean ages ranging from 53.7 to 65.4 years. The predominant histological subtype was serous carcinoma, and **Table 1** shows that FIGO stage III patients outnumbered those at stage IV. Patients in the PDS cohort were treated with IDS directly. The IDS group included patients treated with NACT followed by IDS. The IDS_HIPEC group referred to patients treated with IDS combined with intraoperative HIPEC. In all seven studies, 5–6 mg/mL/min carboplatin and 175 mg/m² paclitaxel were used for NACT, consistent with the 2025 NCCN guidelines (5). Among the three studies incorporating intraoperative HIPEC, two used cisplatin at 75 mg/m², while one used 100 mg/m². Two trials adopted the closed technique, perfused for 90 minutes at 40 °C and 41.5 °C. One trial adopted the open technique, with a 60-minute perfusion at a target temperature of 42 °C to 43 °C (**Table 1**).

**Table 1:** Characteristics of the 7 included studies.

Study	Year	Country	Method	Sample size	Age (Mean ± SD)	FIGO stage				Histologic type		NACT protocol		HIPEC protocol				Design	RER
						Ⅰ	Ⅱ	III	IV	Serous	Others	Drug	Cycles	Drug	Temp (°C)	Duration (min)	Technique		
Lim et al. (15)	2022	South Korea	PDS	49	53.7 ± 10.7	0	0	34	15	41	8	–	–	–	–	–	–	RCT	
			IDS	43	54.4 ± 10.0	0	0	17	26	38	5	Carboplatin 5 mg/mL/min and Paclitaxel 175 mg/m²	3	–	–	–	–		
			IDS**_**HIPEC	34	55.4 ± 13.2	0	0	15	19	32	2			Cisplatin 75 mg/m²	41.5	90	Closed		
Aronson et al. (25)	2023	Netherlands et al.	IDS	123	61.6 ± 7.5	0	0	123	0	107	16	Carboplatin 5–6 mg/mL/min and Paclitaxel 175 mg/m²	At least 3	–	–	–	–	RCT	
			IDS**_**HIPEC	122	60.6 ± 8.3	0	0	122	0	112	10			Cisplatin 100 mg/m²	40	90	Closed		
Cascales Campos et al.	2021	Spain	IDS	36	63.8 ± 8.3	0	0	30	6	NA	NA	Carboplatin 5 mg/mL/min and Paclitaxel 175 mg/m²	3	–	–	–	–	RCT	
			IDS**_**HIPEC	35	55.1 ± 10.9	0	0	33	2	NA	NA			Cisplatin 75 mg/m²	42–43	60	Open		
Onda et al. (23)	2020	Japan	PDS	149	58.4 ± 8.5	0	0	100	49	115	32	–	–	–	–	–	–	RCT	
			IDS	152	60.1 ± 7.4	0	0	105	47	102	28	Carboplatin 6 mg/mL/min and Paclitaxel 175 mg/m²	4	–	–	–	–		
Fagotti et al. (28)	2020	Italy	PDS	84	54.8 ± 9.7	0	0	71	13	82	2	–	–	–	–	–	–	RCT	
			IDS	87	56.2 ± 10.7	0	0	79	8	87	0	Carboplatin 5 mg/mL/min and Paclitaxel 175 mg/m²	3 or 4	–	–	–	–		
Kehoe et al. (27)	2015	UK et al.	PDS	255	65.4 ± 10.8	0	12	190	41	219	36	–	–	–	–	–	–	RCT	
			IDS	219	64.7 ± 9.8	0	7	165	31	185	34	Carboplatin 5–6 mg/mL/min and Paclitaxel 175 mg/m²	3	–	–	–	–		
Vergote et al. (26)	2010	Europe et al.	PDS	336	61.7 ± 10.5	0	0	257	77	220	116	–	–	–	–	–	–	RCT	
			IDS	334	62.7 ± 8.3	0	0	253	81	194	140	Carboplatin 6 mg/mL/min and Paclitaxel 175 mg/m²	3	–	–	–	–		

PDS, primary cytoreductive surgery; NACT, neoadjuvant chemotherapy; IDS, interval cytoreductive surgery; HIPEC, Hyperthermic intraperitoneal chemotherapy; FIGO, Federation of Gynecology and Obstetrics; NA, not available; RCT, randomised controlled trial; RER, references.

### Survival outcomes

#### OS and DFS

**Figure 2:**
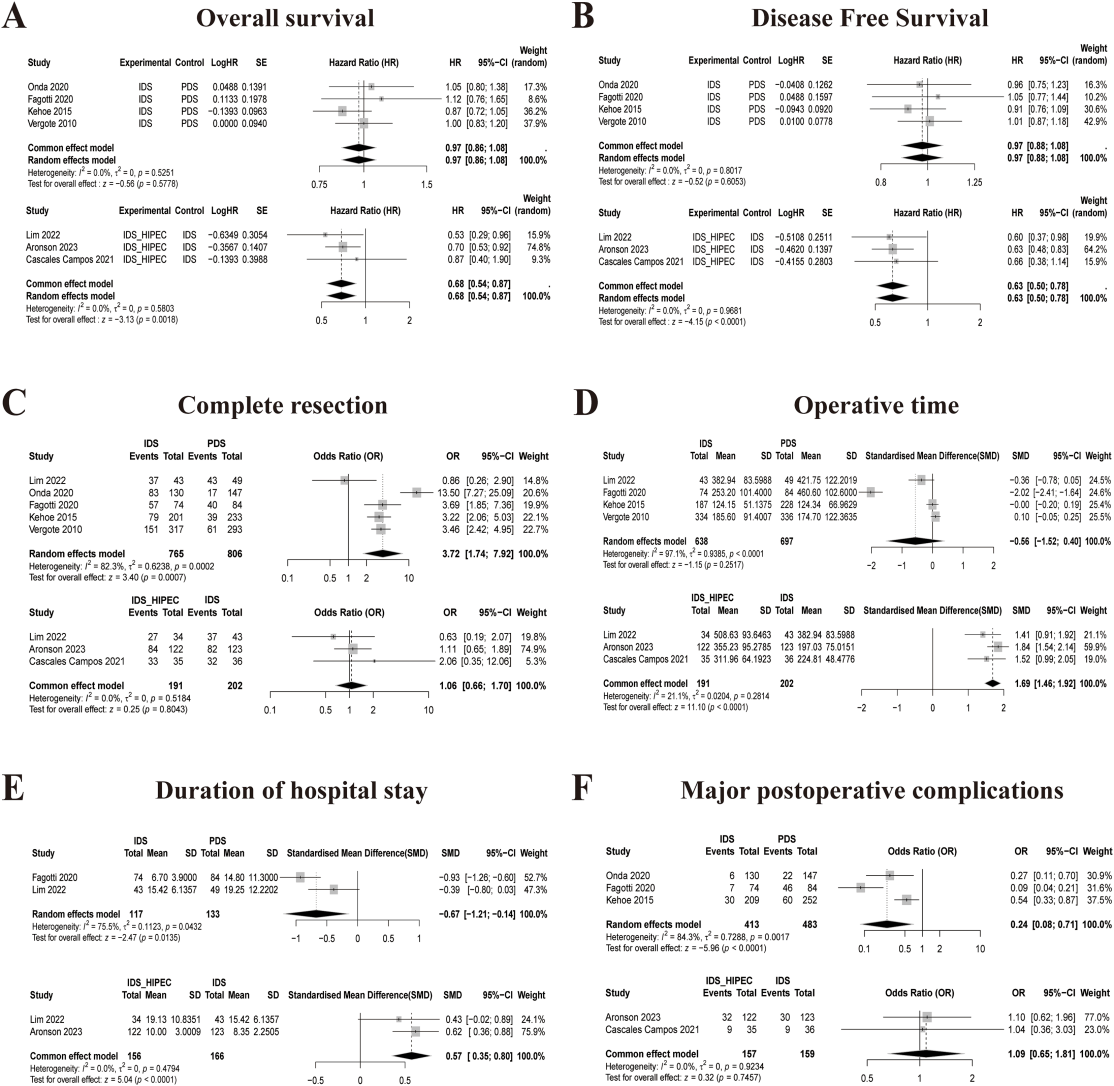
Forest plots comparison of the three treatment strategies for all outcomes. **(A)** Overall survival; **(B)** Disease free survival; **(C)** Complete resection; **(D)** Operative time; **(E)** Duration of hospital stay; **(F)** Major postoperative complications.

**Figure 3:**
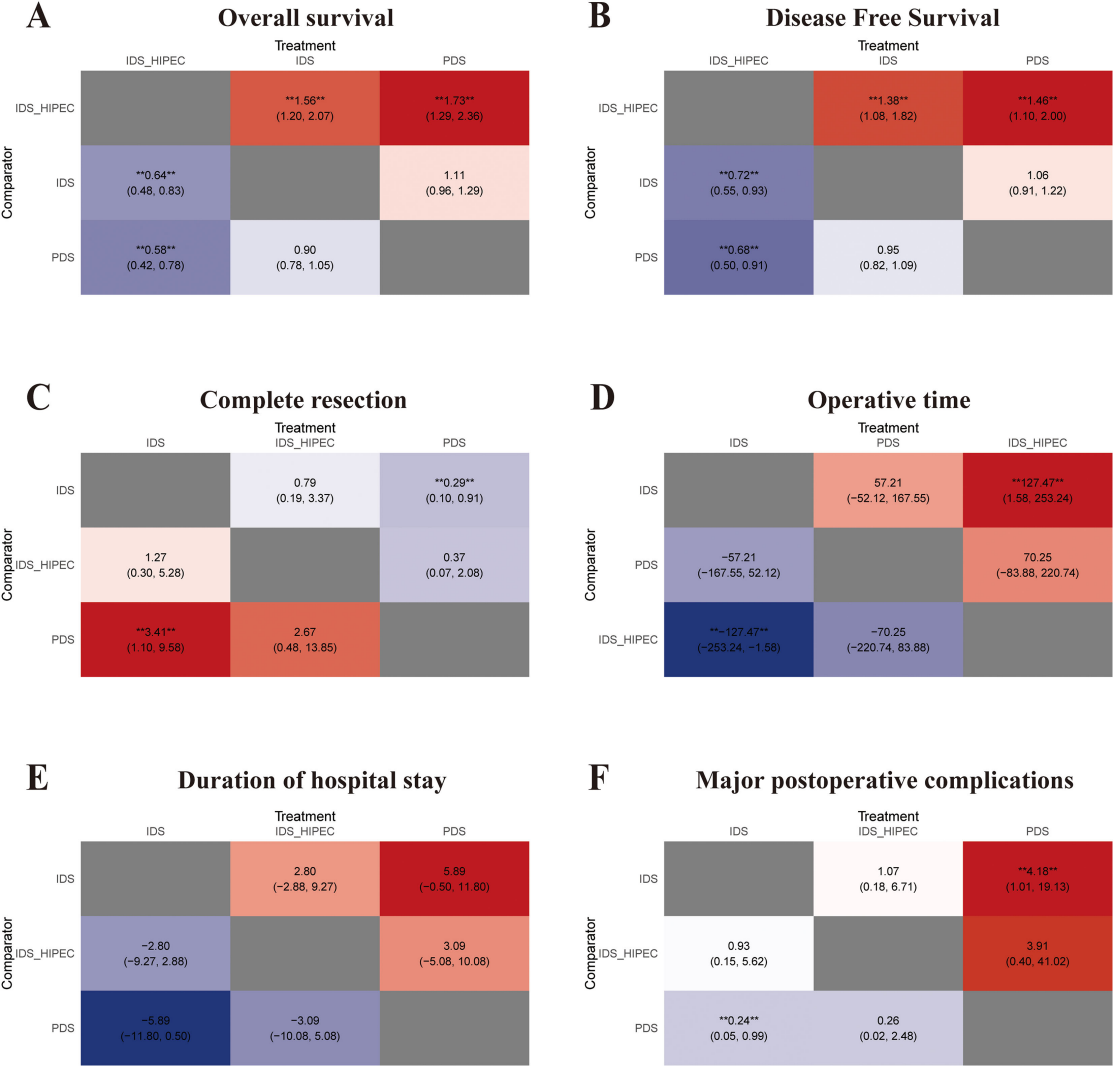
Heat plots of the league table for the three treatment strategies. **(A)** Overall survival; **(B)** Disease free survival; **(C)** Complete resection; **(D)** Operative time; **(E)** Duration of hospital stay; **(F)** Major postoperative complications. “** **” denotes statistical significance, where the confidence interval excludes 1 (for ratios) or excludes 0 (for differences in effects).

**Figure 4:**
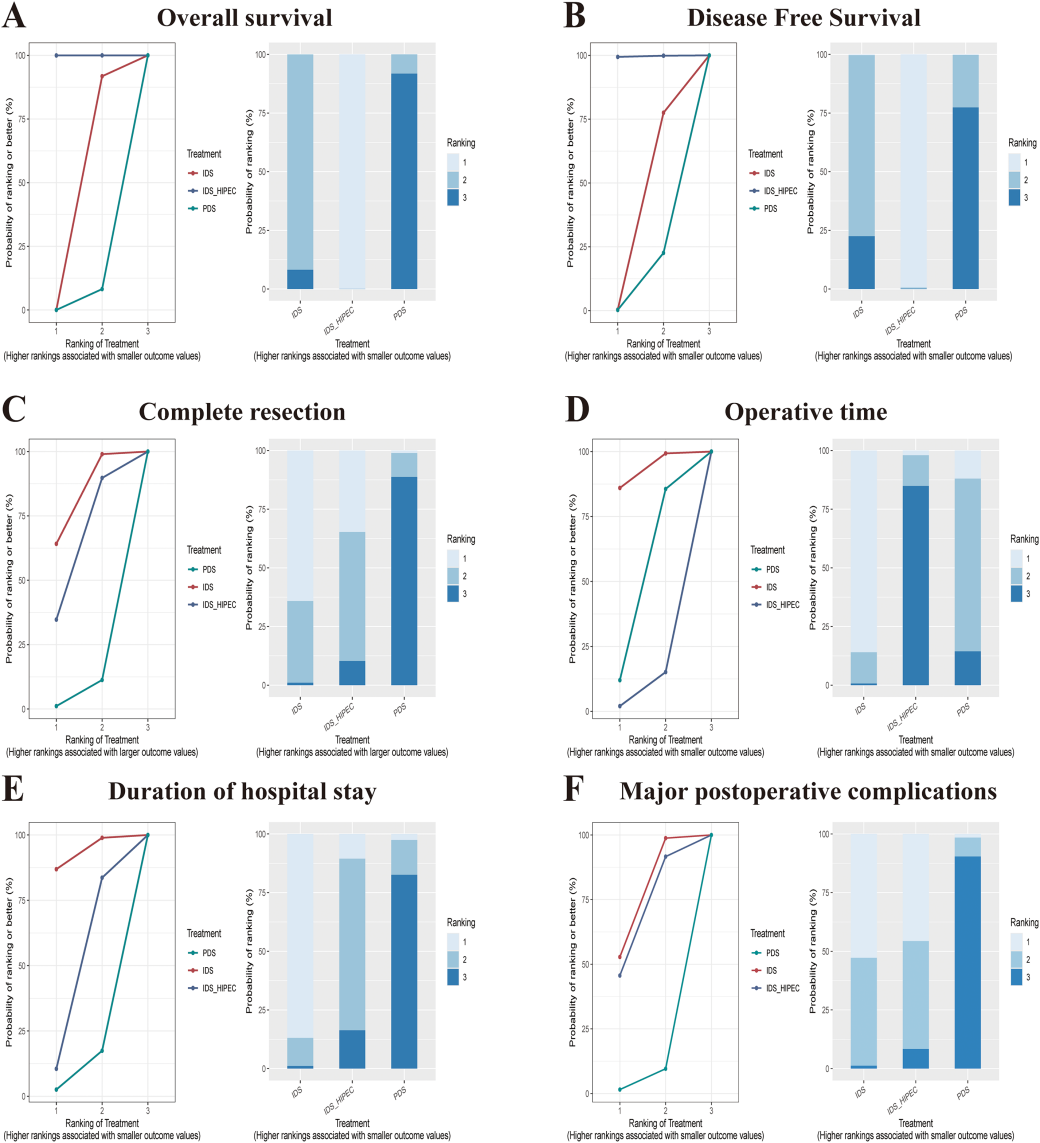
The cumulative ranking plots and nomograms for the three treatment strategies. **(A)** Overall survival; **(B)** Disease free survival; **(C)** Complete resection; **(D)** Operative time; **(E)** Duration of hospital stay; **(F)** Major postoperative complications. PDS, primary debulking surgery; IDS, interval cytoreductive surgery; IDS_HIPEC, interval cytoreductive surgery plus hyperthermic intraperitoneal chemotherapy.

In the PMA (**Figures 2A, B**), the results showed that IDS_HIPEC had longer OS and DFS than IDS (HR = 0.86, 95% CI: 0.54 to 0.87, p = 0.0018; HR = 0.63, 95% CI: 0.50 to 0.78, p < 0.0001). Similarly, in the NMA (**Figures 3A, B**), indirect comparisons indicated that the IDS_HIPEC achieved longer OS and DFS than the PDS (HR = 0.58, 95% CI: 0.42 to 0.78; HR = 0.68, 95% CI: 0.50 to 0.91). Both the PMA and NMA showed that there was no difference between the IDS and PDS in the OS or DFS (**Figures 2A, B****;****3A, B**). IDS_HIPEC ranked the highest probability of benefit in both OS and DFS, with SUCRA values of 0.99 and 0.99, respectively. By contrast, the PDS group exhibited the least advantage in both OS and DFS, with SUCRA values of 0.04 and 0.11, respectively (**Table 2**). The ranking of the three treatment strategies for OS and DFS, according to SUCRA values, is presented in **Figures 4A, B**.

**Table 2:** The surface under the cumulative ranking curve values of all outcomes.

Groups	Overall survival	Disease free survival	Complete resection	Major postoperative complications	Operative time	Duration of hospital stay
PDS	0.04	0.11	0.06	0.05	0.48	0.10
IDS	0.45	0.38	0.81	0.75	0.92	0.99
IDS_HIPEC	0.99	0.99	0.62	0.68	0.08	0.47

PDS, primary debulking surgery; IDS, interval cytoreductive surgery; IDS_HIPEC, interval cytoreductive surgery plus hyperthermic intraperitoneal chemotherapy.

### Strategies efficacy and safety

#### Complete resection

Our study focused solely on the achievement of complete resection following the three treatment strategies. The analysis revealed that the IDS showed superior rates of complete resection compared to the PDS, both in the PMA and NMA (OR = 3.72, 95% CI: 1.74 to 7.92, p = 0.0007; OR = 3.41, 95% CI: 1.10 to 9.58; **Figures 2C**, **3C**, **4C**). The SUCRA values for complete resection in the NMA were 0.06 for PDS, 0.81 for IDS, and 0.62 for IDS_HIPEC (**Table 2**).

#### Operative time

Operative time was reported in six studies involving 1,685 patients across the three treatment strategies. Intraoperative HIPEC necessitated additional time during IDS, resulting in IDS_HIPEC having the lowest SUCRA value of 0.08 (**Table 2**). No significant statistical difference in operative time was observed between the IDS and PDS. Based on SUCRA values, IDS was associated with the shortest operative time, with a SUCRA value of 0.92, a trend consistently observed both in the PMA and the ranking plot (**Figures 2D**, **3D**, **4G**).

#### Duration of hospital stay

According to the PMA (**Figure 2E**), hospital stay was significantly reduced in the IDS group compared with the PDS (MD = -0.67, 95% CI: -1.21 to -0.14, p = 0.0135) and the IDS_HIPEC (MD = 0.57, 95% CI: 0.35 to 0.80, p < 0.0001). In this study, the IDS achieved a SUCRA value of 0.99 for hospital stay, suggesting that NACT before cytoreductive surgery effectively reduces hospitalization duration (Figures **3E**, **4E**). The SUCRA values for the remaining strategies are summarized in **Table 2**.

#### Major postoperative complications

We compared the incidence of grade 3 and 4 postoperative adverse events among the three treatment strategies. Both the PMA (**Figure 2F**) and NMA (**Figure 3F**) demonstrated that IDS significantly reduced the incidence of major postoperative complications compared with direct PDS for advanced OC (OR = 0.24, 95% CI: 0.08 to 0.71, p < 0.0001; OR = 0.24, 95% CI: 0.05 to 0.99). Our study showed that the addition of intraoperative HIPEC to IDS did not result in a higher incidence of postoperative major complications. Notably, IDS showed the most significant advantage in reducing postoperative major complications. The SUCRA value for IDS was 0.75, whereas the SUCRA value for PDS was only 0.05 (**Table 2**). The ranking plot (**Figure 4F**) also revealed similar results.

## Discussion

This study is the first NMA to investigate the efficacy and safety of three different treatment strategies for patients with primary advanced OC. To strengthen the credibility of our results, we restricted the population to patients with primary advanced OC, and only high-quality RCTs were included. By carefully scrutinizing the methods sections of each original study, we sought to minimize the potential influence of inter-study heterogeneity on our conclusions. We assessed survival outcome via the OS and DFS, while a comprehensive synthesis was performed for complete resection, postoperative major complications, operative time, and duration of hospital stay across the different treatment strategies.

First, the wide time span of the studies included in this study is limited by the medical standards of the respective eras. Combined with the objective differences that exist in the quality of care across different research centers, these confounding factors may limit the applicability of our findings. As shown in **Table 1**, even when adhering to guidelines, the HIPEC regimens in the included studies exhibited differences regarding drug, temperature, duration, and technique. While surgical expertise and institutional volume are critical determinants in the management of advanced OC, variability in surgical quality control and center volume across the trials may have contributed to heterogeneity in complete resection rates and complication profiles. Moreover, the allocation of patients relied heavily on assessments of tumor resectability and preoperative performance status, a process that inevitably introduces selection bias. Since FIGO stage IV disease predicts a worse survival outcome, and an imbalance in the number of patients with FIGO stage III and FIGO stage IV disease was observed, this may affect the accurate assessment of the survival benefits of different treatment strategies.

OS and DFS are the key survival endpoints used to assess therapeutic outcomes in advanced OC patients. For patients with primary advanced OC, adding HIPEC during IDS provides significant survival benefits, prolonging both OS and DFS. Multiple meta-analyses have reported findings consistent with ours (29, 30). Our hypothesis is that after NACT reduces tumor load, HIPEC exerts its thermal effects and delivers high-concentration chemotherapy directly within the peritoneal cavity, enabling enhanced eradication of microscopic residual lesions, controlling micrometastases, and providing additional survival benefits. Furthermore, studies employing transcriptome sequencing before and after HIPEC in advanced OC have revealed that HIPEC reshapes the tumor immune microenvironment, thereby impacting patient outcomes (31, 32). Results from a multicenter retrospective cohort study indicated that for stage III epithelial OC, HIPEC combined with PDS improved long-term OS exceeding 5 years (33). However, a single-center retrospective study by Ghirardi et al. and a subgroup analysis by Lim et al. both showed no survival benefit of adding HIPEC to PDS (13, 34). Unfortunately, no RCT has yet been published evaluating HIPEC directly combined with PDS in primary advanced OC, and its survival benefit remains uncertain. As this study did not include direct comparative studies between PDS and IDS_HIPEC, the conclusions derived from indirect comparisons through NMA should be interpreted with caution. Larger multicenter randomized trials are still needed to further confirm the value of PDS combined with HIPEC.

Although the SUCRA value of IDS was higher than that of PDS, the difference did not reach statistical significance. IDS did not provide additional OS or DFS benefits for patients with advanced OC, which is consistent with many previous studies (17, 35, 36). Our hypothesis is that in the included studies, PDS was able to remove most of the tumor at an early stage but due to widespread tumor masses, complete resection was often unattainable. So a higher burden of residual lesions after PDS may lead to rapid tumor progression. While NACT improved the complete resection rate, it delayed cytoreduction time and may reduce postoperative platinum sensitivity. The ASCO (American Society of Clinical Oncology) meeting reported a recent RCT comparing PDS and IDS for patients with primary advanced OC (37). The results showed that in accredited high-quality surgical centers, PDS significantly prolonged median PFS and showed a trend toward improved OS in primary advanced OC patients with good performance status, compared to IDS. The study concluded that when similar R0 rates were achieved (62.9% in PDS; 76.6% in IDS), PDS was associated with better survival outcomes compared to IDS. Since the R0 rates for PDS and IDS in the included studies were not similar, the lack of a statistically significant difference in survival benefit between PDS and IDS requires cautious interpretation. Moreover, in clinical decision-making, the choice between IDS and PDS requires a detailed assessment of performance status, tumor burden, and the proficiency of the treatment center to ensure careful selection, aiming to achieve R0 resection feasibility for better survival benefits.

As this study is unable to directly evaluate the absolute survival differences among the three treatment strategies for primary advanced OC, and given the inherent uncertainty associated with indirect survival estimates based on the transitivity assumption, the conclusions regarding survival benefits warrant cautious interpretation. It is also hoped that more high quality studies will be conducted in the future to investigate these absolute survival differences.

The amount of residual disease in the abdominal cavity at the end of surgery is the most important prognostic factor for patients with advanced OC (38). Our study showed that IDS markedly enhanced the complete resection rate (SUCRA = 0.81), which was in agreement with other studies (17, 36). A prospective study by Mlees et al. indicated that for patients with advanced OC, IDS can result in better pathological responses and surgical resection outcomes, thereby improving OS and PFS (39). NACT can reduce tumor volume, alleviate ascites, decrease adhesions between tumor tissue and surrounding structures, and improve surgical feasibility, thereby increasing the complete resection rate. For advanced OC patients with impaired physical status, multiple comorbidities, or a low probability of complete cytoreduction, the appropriate preoperative use of NACT is crucial to increase CRS feasibility and raise complete resection rates. HIPEC does not directly affect the complete resection rate, while on the basis of complete resection, HIPEC could further eradicate microscopic lesions, extending the no gross tumor status to the microscopic dimension.

The operation time and duration of hospital stay are key outcome measures. Because HIPEC is performed after the completion of surgery, it does not add additional complexity to the procedure. Although the IDS_HIPEC had the longest operative time, the extra time was relatively fixed and controllable, so it did not represent an absolute strategic disadvantage. Our results show that IDS could reduce operative time (SUCRA = 0.92). We hypothesize that NACT reduces tumor size and decreases adhesion between tumor tissue and surrounding structures, significantly lowering surgical difficulty and shortening procedure duration. However, this conclusion requires further validation through additional research. Due to shorter operative times and fewer major postoperative complications, patients receiving NACT typically recover and leave the hospital earlier (SUCRA = 0.99). A retrospective study revealed that patients who underwent IDS had a significantly shorter duration of stay in the intensive care unit, but there was no substantial difference in the overall duration of hospital stay (40). The addition of intraoperative HIPEC did not materially prolong the duration of hospital stay (SUCRA = 0.47), which is consistent with other studies (24).

Postoperative complications directly reflect the effectiveness and safety of treatment strategies. Across the included studies, major postoperative complications primarily encompassed hemorrhage, infection, venous thromboembolism, ileus, and others. Our results indicate that PDS had a higher incidence of major postoperative complications (SUCRA = 0.05). High tumor burden, involvement of multiple organs, and poor preoperative physical condition may collectively increase the difficulty of PDS and elevate the risk of complications. Conversely, a study has reported that patients undergoing IDS experience a significantly reduced proportion of organs requiring resection, and distant metastatic lesions are also less frequently removed (23). In our study, IDS significantly reduced major postoperative complications (SUCRA = 0.75). Furthermore, in our analysis, the combination of IDS and HIPEC did not noticeably increase major postoperative complications (SUCRA = 0.68). Similarly, Marzuqi et al. found that HIPEC does not increase major postoperative complications, though minor adverse events such as electrolyte disorders, leukopenia, and nausea persist (41). Taliento et al. noted that HIPEC treatment may be associated with a higher incidence of acute kidney injury (42). Therefore, HIPEC should be performed in qualified institutions under strictly standardized protocols.

This study has several limitations. First, the number of available RCTs was limited, with only seven RCTs meeting the inclusion criteria; therefore, the conclusions drawn from this study should be interpreted with caution. Second, this study only included high-grade serous carcinoma, and the safety and efficacy of the three treatments for other OC subtypes are still unclear. Finally, information on subsequent therapies administered to patients at recurrence or progression was limited. Therefore, the potential impact of introducing biological agents such as polymerase inhibitors or bevacizumab on survival outcomes remains uncertain, and further clinical studies involving larger populations are needed to validate these conclusions.

## Conclusions

Until new evidence becomes available, IDS plus intraoperative HIPEC are associated with better OS and DFS for patients with primary advanced OC. Although HIPEC extended the operative time, it did not increase major postoperative complications or lengthen the duration of hospital stay. IDS had advantages in increasing the complete resection rate and reducing major postoperative complications.

## Data Availability

The raw data supporting the conclusions of this article will be made available by the authors, without undue reservation.
