# Supplementary material for: Comparative efficacy and safety of different treatment strategies for primary advanced ovarian cancer: a systematic review and network meta-analysis of randomized control trials
Source: Front Oncol. 2026 Mar 12;16:1757826. doi: 10.3389/fonc.2026.1757826 (PMC13018150; doi:10.3389/fonc.2026.1757826)

Supplementary Figure 1

	Random sequence generation (selection bias)	Allocation concealment (selection bias)	Blinding of participants and personnel (performance bias)	Blinding of outcome assessment (detection bias)	Incomplete outcome data (attrition bias)	Selective reporting (reporting bias)	Other bias
Antonio 2021	+	?	?	?	?	?	?
Aronson 2023	+	+	?	?	?	+	?
Fagotti 2020	+	+	?	?	?	?	?
Kehoe 2015	+	+	?	?	+	?	?
Lim 2022	+	+	?	?	?	+	?
Onda 2020	+	+	?	?	+	+	?
Vergote 2010	+	?	?	?	?	?	?

Supplementary Figure 2

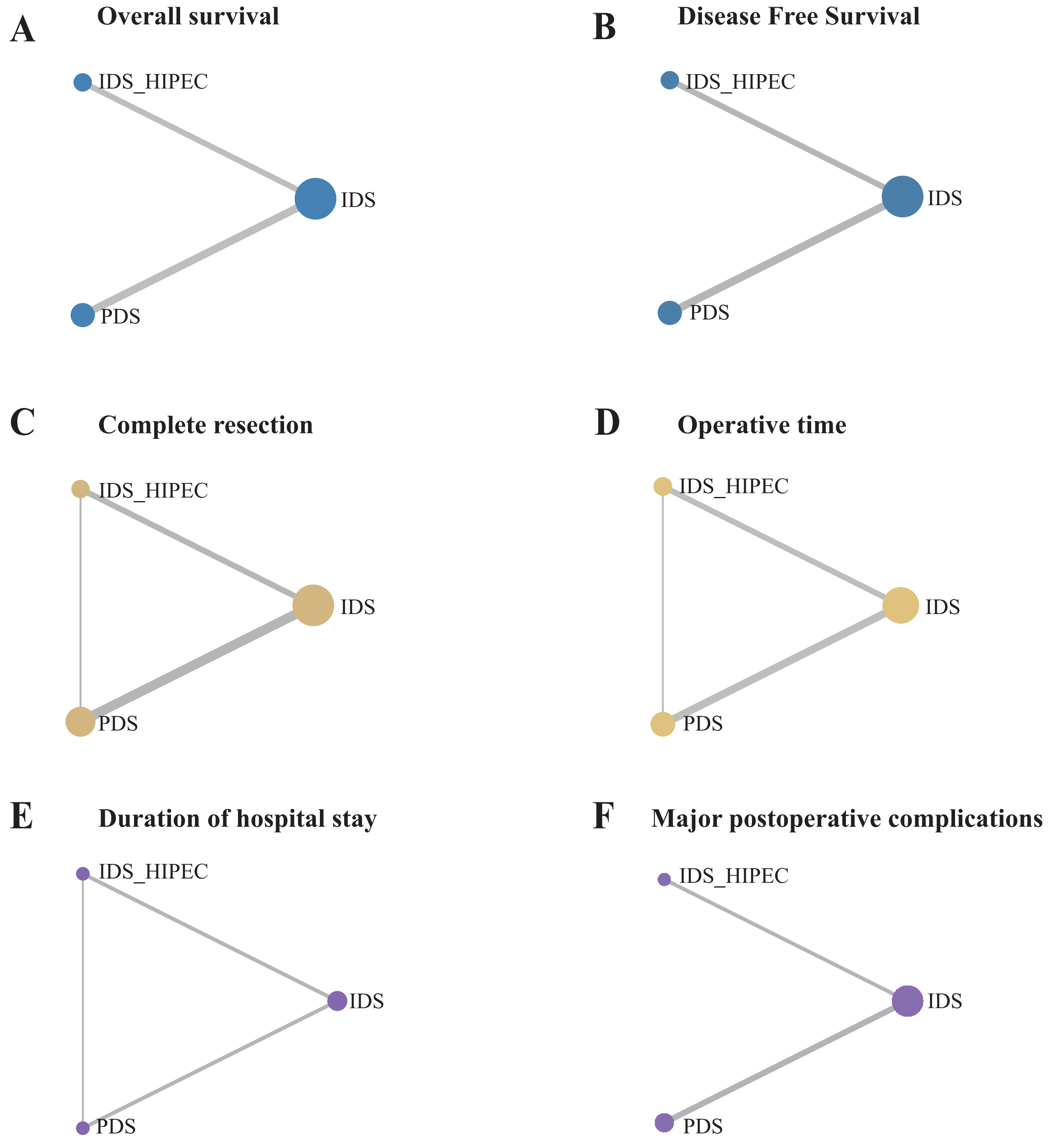

Supplementary Figure 3

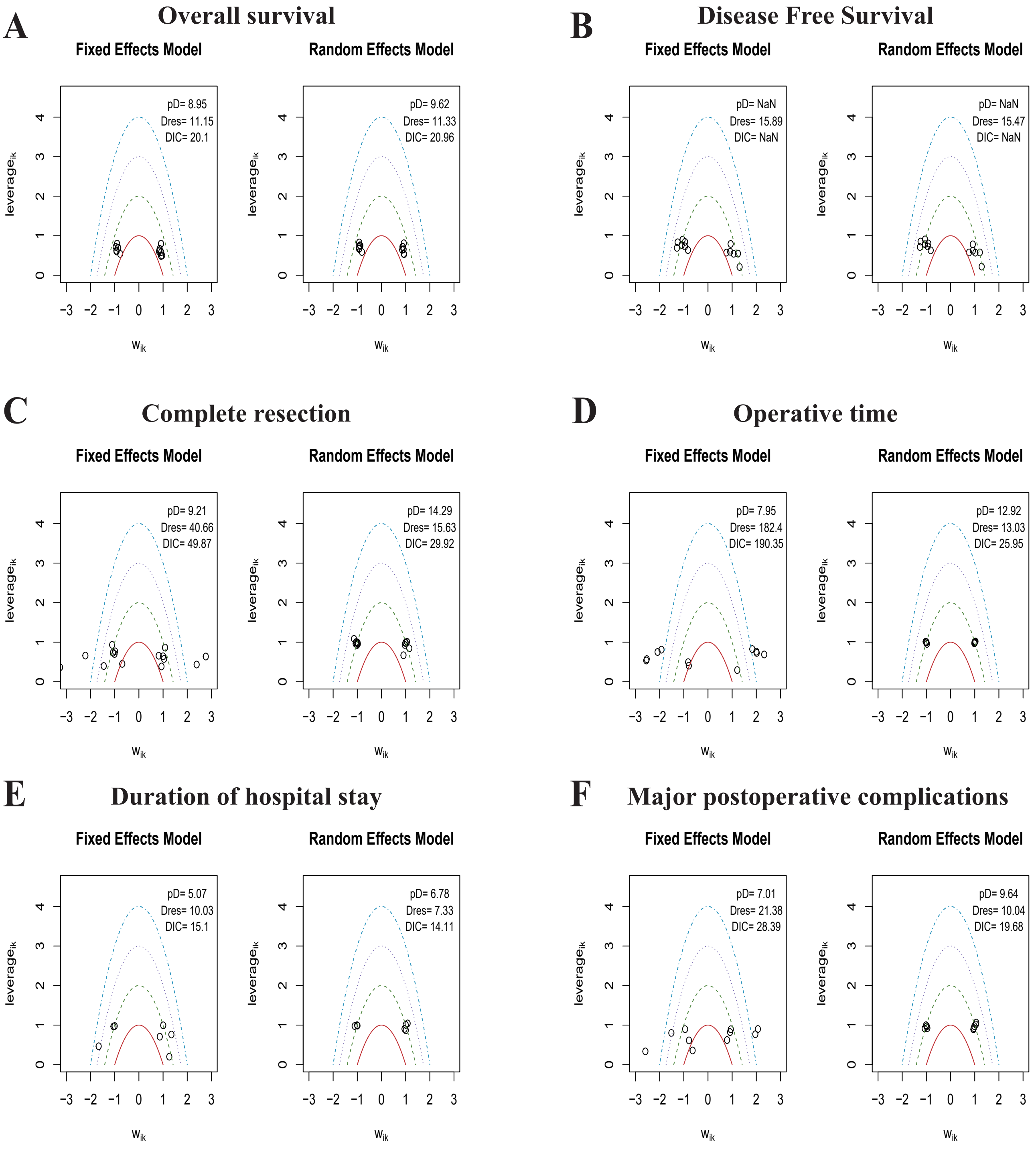

Supplementary Figure 4

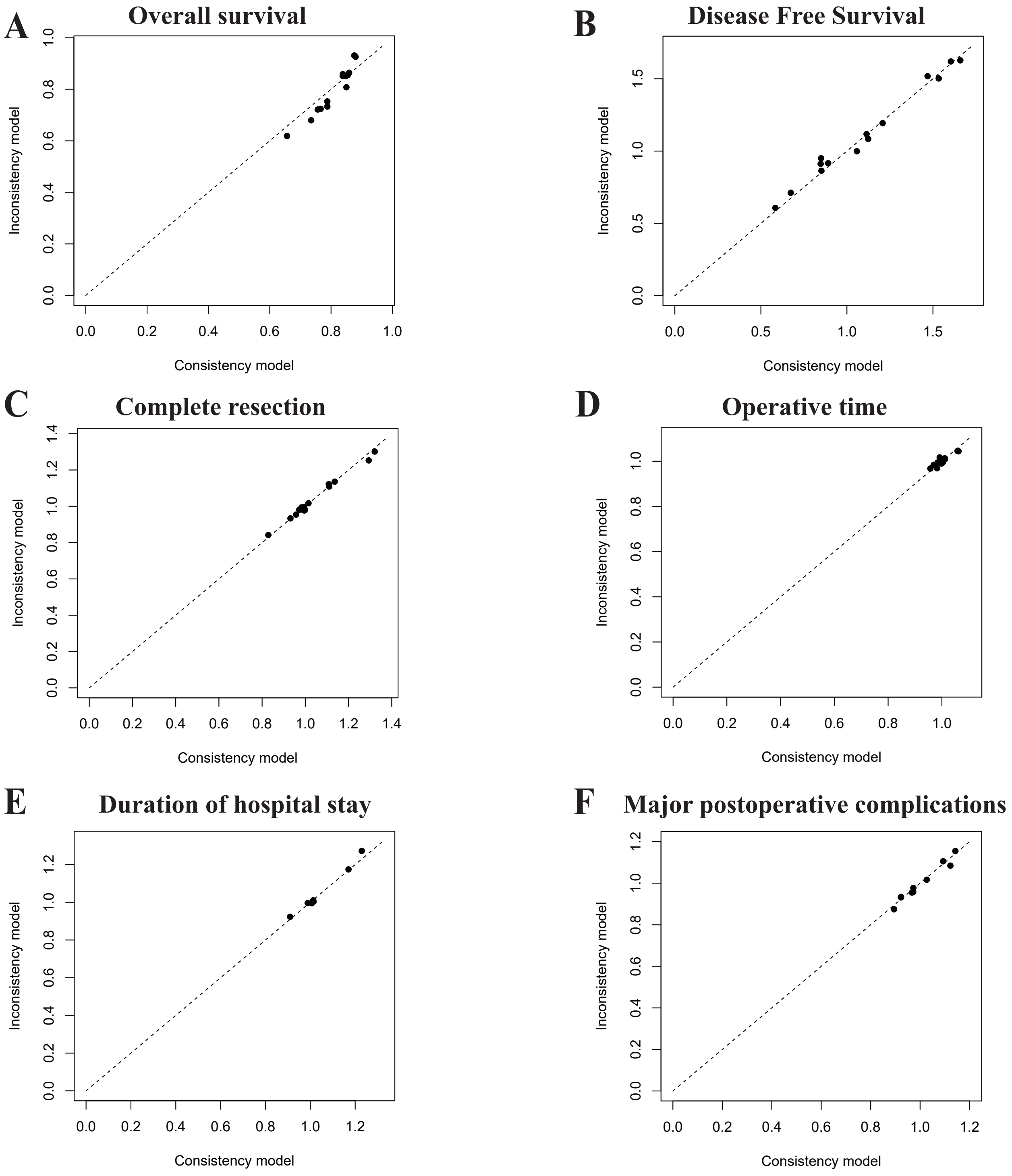

Supplementary Figure 5

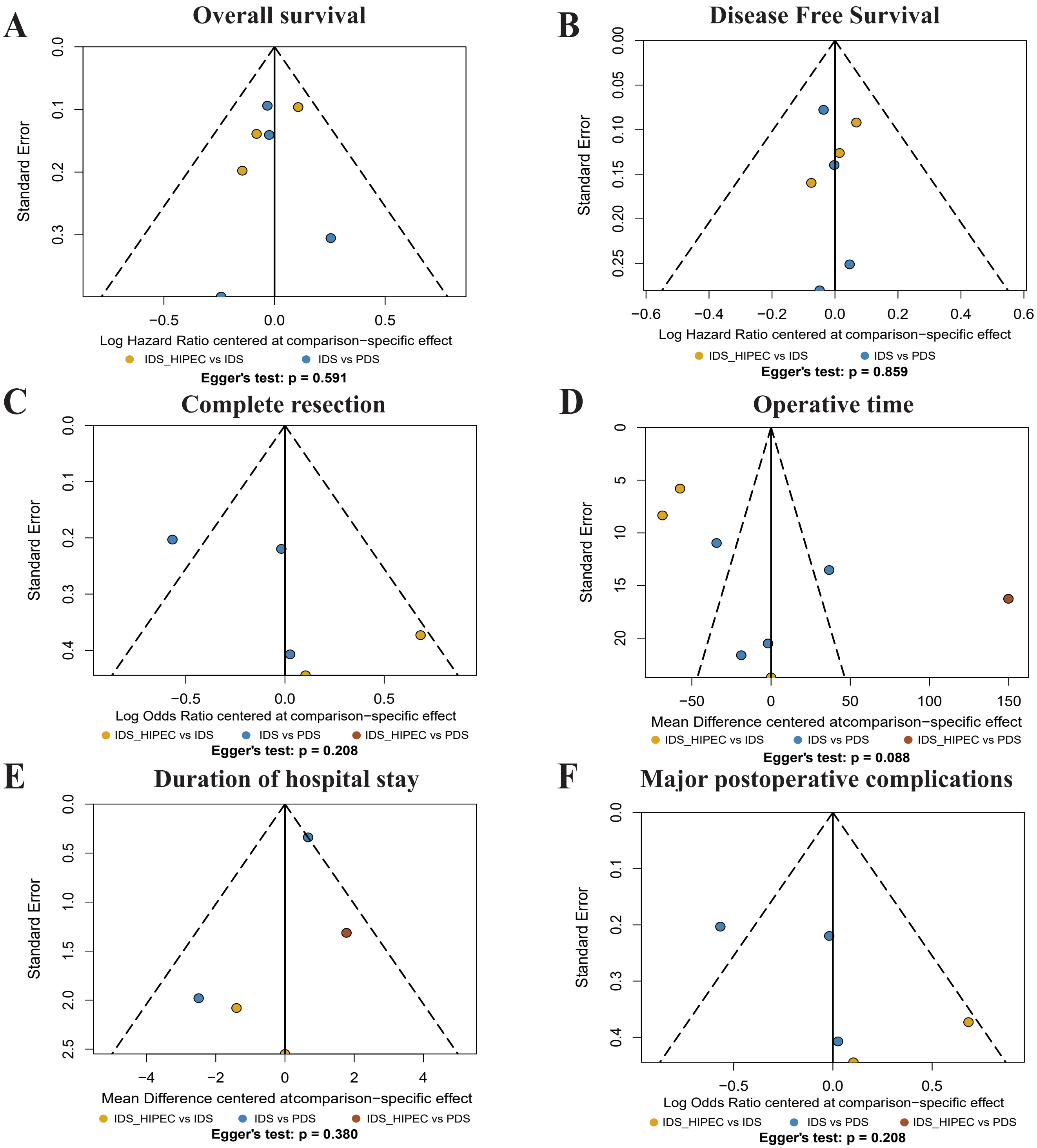

Supplement: Supplementary file 1 [file DataSheet1.pdf]
